# Supplementary material for: A single-cell atlas of the cycling murine ovary
Source: eLife. 2022 Oct 7;11:e77239. doi: 10.7554/eLife.77239 (PMC9545525; doi:10.7554/eLife.77239)
Supplement: Supplementary file 2. [file elife-77239-supp2.docx]

**Supplementary File 2 - Top 10 markers expressed in each ovary cluster**

| **Granulosa** | avg_log2FC | **Mesenchyme** | avg_log2FC | **Endothelium** | avg_log2FC |
| --- | --- | --- | --- | --- | --- |
| Nr5a2 (1) | 2.37 | Col1a2 (2) | 2.80 | Kdr (3) | 3.21 |
| Bex4 (4) | 2.08 | Col3a1 (5) | 2.68 | Mmrn2 (6) | 3.15 |
| Lect1 (7) | 1.95 | Col1a1 (4) | 2.64 | Cdh5 (4) | 3.02 |
| Fst (8) | 1.90 | Ogn (9,10) | 2.40 | Egfl7(11) | 2.93 |
| Hsd17b1 (12) | 1.89 | Bgn (13) | 2.37 | Pecam1 (14) | 2.91 |
| Slc18a2 (15,16) | 1.87 | Tcf21 (17) | 2.35 | Cldn5 (18) | 2.87 |
| Inha (15) | 1.82 | Dcn (19) | 2.34 | Esam (20) | 2.84 |
| Serpine2 (21) | 1.76 | Pdgfra (22) | 2.30 | Flt1 (23) | 2.83 |
| Ivns1abp (24) | 1.65 | Lum (10) | 2.18 | Cd93 (6,25) | 2.79 |
| Fam13a | 1.59 | Mgp (26) | 2.17 | Ctla2a (27) | 2.77 |

| **Immune** | avg_log2FC | **Oocyte** | avg_log2FC | **Epithelilum** | avg_log2FC |
| --- | --- | --- | --- | --- | --- |
| Lyz2 (28) | 3.62 | Gm15698 | 4.06 | Upk1b (29) | 3.23 |
| Laptm5 (30) | 3.54 | Gdf9 (31) | 3.91 | Upk3b (32) | 3.17 |
| H2Aa (33) | 3.48 | H1foo (34) | 3.78 | Lgals7 (35) | 3.09 |
| Cd74 (36) | 3.47 | Padi6 (37) | 3.78 | Aldh1a2 (38) | 3.04 |
| Fcer1g (39) | 3.45 | Ooep (40) | 3.75 | Mt2 (41,42) | 2.95 |
| Ctss (36) | 3.37 | Oosp1 (43) | 3.72 | Ildr2 (44) | 2.90 |
| H2.Eb1 (45) | 3.33 | Rfpl4 (46) | 3.62 | Krt18 (47) | 2.82 |
| C1qb (48) | 3.32 | Tcl1 (43) | 3.56 | Gpm6a | 2.76 |
| C1qc (48) | 3.23 | Khdc1b (49) | 3.54 | Plxna4 (50) | 2.75 |
| Cd52 | 3.17 | Nlrp14 (51) | 3.53 | Krt7(47) | 2.67 |

**Bibliography**

1. **Meinsohn M-C, Morin F, Bertolin K, Duggavathi R, Schoonjans K, Murphy BD.** The Orphan Nuclear Receptor Liver Homolog Receptor-1 (Nr5a2) Regulates Ovarian Granulosa Cell Proliferation. *J Endocr Soc* 2018;2(1):24–41.

2. **Hodgkinson K, Forrest LA, Vuong N, Garson K, Djordjevic B, Vanderhyden BC.** GREB1 is an estrogen receptor-regulated tumour promoter that is frequently expressed in ovarian cancer. *Oncogene* 2018;37(44):5873–5886.

3. **Terman BI, Dougher-Vermazen M, Carrion ME, Dimitrov D, Armellino DC, Gospodarowicz D, Böhlen P.** Identification of the KDR tyrosine kinase as a receptor for vascular endothelial cell growth factor. *Biochem Biophys Res Commun* 1992;187(3):1579–1586.

4. **Wagner M, Yoshihara M, Douagi I, Damdimopoulos A, Panula S, Petropoulos S, Lu H, Pettersson K, Palm K, Katayama S, Hovatta O, Kere J, Lanner F, Damdimopoulou P.** Single-cell analysis of human ovarian cortex identifies distinct cell populations but no oogonial stem cells. *Nat Commun* 2020;11(1):1147.

5. **Parikh A, Lee C, Joseph P, Marchini S, Baccarini A, Kolev V, Romualdi C, Fruscio R, Shah H, Wang F, Mullokandov G, Fishman D, D’Incalci M, Rahaman J, Kalir T, Redline RW, Brown BD, Narla G, DiFeo A.** microRNA-181a has a critical role in ovarian cancer progression through the regulation of the epithelial-mesenchymal transition. *Nat Commun* 2014;5:2977.

6. **Galvagni F, Nardi F, Spiga O, Trezza A, Tarticchio G, Pellicani R, Andreuzzi E, Caldi E, Toti P, Tosi GM, Santucci A, Iozzo RV, Mongiat M, Orlandini M.** Dissecting the CD93-Multimerin 2 interaction involved in cell adhesion and migration of the activated endothelium. *Matrix Biol* 2017;64:112–127.

7. **Richards JS, Fan H-Y, Liu Z, Tsoi M, Laguë M-N, Boyer A, Boerboom D.** Either Kras activation or Pten loss similarly enhance the dominant-stable CTNNB1-induced genetic program to promote granulosa cell tumor development in the ovary and testis. *Oncogene* 2012;31(12):1504–1520.

8. **Zhou Q, Wan M, Wei Q, Song Q, Xiong L, Huo J, Huang J.** Expression, Regulation, and Functional Characterization of FST Gene in Porcine Granulosa Cells. *Anim Biotechnol* 2016;27(4):295–302.

9. **Hummitzsch K, Hatzirodos N, Macpherson AM, Schwartz J, Rodgers RJ, Irving-Rodgers HF.** Transcriptome analyses of ovarian stroma: tunica albuginea, interstitium and theca interna. *Reproduction* 2019;157(6):545–565.

10. **Hartanti MD, Hummitzsch K, Irving-Rodgers HF, Bonner WM, Copping KJ, Anderson RA, McMillen IC, Perry VEA, Rodgers RJ.** Morphometric and gene expression analyses of stromal expansion during development of the bovine fetal ovary. *Reprod Fertil Dev* 2019;31(3):482–495.

11. **Bambino K, Lacko LA, Hajjar KA, Stuhlmann H.** Epidermal growth factor-like domain 7 is a marker of the endothelial lineage and active angiogenesis. *Genesis* 2014;52(7):657–670.

12. **He W, Gauri M, Li T, Wang R, Lin S-X.** Current knowledge of the multifunctional 17β-hydroxysteroid dehydrogenase type 1 (HSD17B1). *Gene* 2016;588(1):54–61.

13. **Oksjoki S, Sallinen S, Vuorio E, Anttila L.** Cyclic expression of mRNA transcripts for connective tissue components in the mouse ovary. *Mol Hum Reprod* 1999;5(9):803–808.

14. **Park S, DiMaio TA, Scheef EA, Sorenson CM, Sheibani N.** PECAM-1 regulates proangiogenic properties of endothelial cells through modulation of cell-cell and cell-matrix interactions. *Am J Physiol Cell Physiol* 2010;299(6):C1468-1484.

15. **Meinsohn M-C, Saatcioglu HD, Wei L, Li Y, Horn H, Chauvin M, Kano M, Nguyen NMP, Nagykery N, Kashiwagi A, Samore WR, Wang D, Oliva E, Gao G, Morris ME, Donahoe PK, Pépin D.** Single-cell sequencing reveals suppressive transcriptional programs regulated by MIS/AMH in neonatal ovaries. *Proc Natl Acad Sci U S A* 2021;118(20). doi:10.1073/pnas.2100920118.

16. **Wigglesworth K, Lee K-B, Emori C, Sugiura K, Eppig JJ.** Transcriptomic diversification of developing cumulus and mural granulosa cells in mouse ovarian follicles. *Biol Reprod* 2015;92(1):23.

17. **Varankar SS, More M, Abraham A, Pansare K, Kumar B, Narayanan NJ, Jolly MK, Mali AM, Bapat SA.** Functional balance between Tcf21-Slug defines cellular plasticity and migratory modalities in high grade serous ovarian cancer cell lines. *Carcinogenesis* 2020;41(4):515–526.

18. **Herr D, Fraser HM, Konrad R, Holzheu I, Kreienberg R, Wulff C.** Human chorionic gonadotropin controls luteal vascular permeability via vascular endothelial growth factor by down-regulation of a cascade of adhesion proteins. *Fertil Steril* 2013;99(6):1749–1758.

19. **Hatzirodos N, Hummitzsch K, Irving-Rodgers HF, Rodgers RJ.** Transcriptome comparisons identify new cell markers for theca interna and granulosa cells from small and large antral ovarian follicles. *PLoS One* 2015;10(3):e0119800.

20. **Angelos MG, Abrahante JE, Blum RH, Kaufman DS.** Single Cell Resolution of Human Hematoendothelial Cells Defines Transcriptional Signatures of Hemogenic Endothelium. *Stem Cells* 2018;36(2):206–217.

21. **Bédard J, Brûlé S, Price CA, Silversides DW, Lussier JG.** Serine protease inhibitor-E2 (SERPINE2) is differentially expressed in granulosa cells of dominant follicle in cattle. *Mol Reprod Dev* 2003;64(2):152–165.

22. **Sleer LS, Taylor CC.** Cell-type localization of platelet-derived growth factors and receptors in the postnatal rat ovary and follicle. *Biol Reprod* 2007;76(3):379–390.

23. **de Vries C, Escobedo JA, Ueno H, Houck K, Ferrara N, Williams LT.** The fms-like tyrosine kinase, a receptor for vascular endothelial growth factor. *Science* 1992;255(5047):989–991.

24. **Wu Y, Lin J, Li X, Han B, Wang L, Liu M, Huang J.** Transcriptome profile of one-month-old lambs’ granulosa cells after superstimulation. *Asian-Australas J Anim Sci* 2017;30(1):20–33.

25. **Galvagni F, Nardi F, Maida M, Bernardini G, Vannuccini S, Petraglia F, Santucci A, Orlandini M.** CD93 and dystroglycan cooperation in human endothelial cell adhesion and migration adhesion and migration. *Oncotarget* 2016;7(9):10090–10103.

26. **Sterzyńska K, Klejewski A, Wojtowicz K, Świerczewska M, Andrzejewska M, Rusek D, Sobkowski M, Kędzia W, Brązert J, Nowicki M, Januchowski R.** The Role of Matrix Gla Protein (MGP) Expression in Paclitaxel and Topotecan Resistant Ovarian Cancer Cell Lines. *Int J Mol Sci* 2018;19(10). doi:10.3390/ijms19102901.

27. **Kalucka J, de Rooij LPMH, Goveia J, Rohlenova K, Dumas SJ, Meta E, Conchinha NV, Taverna F, Teuwen L-A, Veys K, García-Caballero M, Khan S, Geldhof V, Sokol L, Chen R, Treps L, Borri M, de Zeeuw P, Dubois C, Karakach TK, Falkenberg KD, Parys M, Yin X, Vinckier S, Du Y, Fenton RA, Schoonjans L, Dewerchin M, Eelen G, Thienpont B, Lin L, Bolund L, Li X, Luo Y, Carmeliet P.** Single-Cell Transcriptome Atlas of Murine Endothelial Cells. *Cell* 2020;180(4):764-779.e20.

28. **Cochain C, Vafadarnejad E, Arampatzi P, Pelisek J, Winkels H, Ley K, Wolf D, Saliba A-E, Zernecke A.** Single-Cell RNA-Seq Reveals the Transcriptional Landscape and Heterogeneity of Aortic Macrophages in Murine Atherosclerosis. *Circ Res* 2018;122(12):1661–1674.

29. **Carpenter AR, Becknell MB, Ching CB, Cuaresma EJ, Chen X, Hains DS, McHugh KM.** Uroplakin 1b is critical in urinary tract development and urothelial differentiation and homeostasis. *Kidney Int* 2016;89(3):612–624.

30. **Glowacka WK, Alberts P, Ouchida R, Wang J-Y, Rotin D.** LAPTM5 protein is a positive regulator of proinflammatory signaling pathways in macrophages. *J Biol Chem* 2012;287(33):27691–27702.

31. **Paulini F, Melo EO.** The role of oocyte-secreted factors GDF9 and BMP15 in follicular development and oogenesis. *Reprod Domest Anim* 2011;46(2):354–361.

32. **Rudat C, Grieskamp T, Röhr C, Airik R, Wrede C, Hegermann J, Herrmann BG, Schuster-Gossler K, Kispert A.** Upk3b is dispensable for development and integrity of urothelium and mesothelium. *PLoS One* 2014;9(11):e112112.

33. **Yang P, Wu Q, Sun L, Fang P, Liu L, Ji Y, Park J-Y, Qin X, Yang X, Wang H.** Adaptive Immune Response Signaling Is Suppressed in Ly6C(high) Monocyte but Upregulated in Monocyte Subsets of ApoE (-/-) Mice - Functional Implication in Atherosclerosis. *Front Immunol* 2021;12:809208.

34. **Tanaka M, Kihara M, Hennebold JD, Eppig JJ, Viveiros MM, Emery BR, Carrell DT, Kirkman NJ, Meczekalski B, Zhou J, Bondy CA, Becker M, Schultz RM, Misteli T, De La Fuente R, King GJ, Adashi EY.** H1FOO is coupled to the initiation of oocytic growth. *Biol Reprod* 2005;72(1):135–142.

35. **Sewgobind NV, Albers S, Pieters RJ.** Functions and Inhibition of Galectin-7, an Emerging Target in Cellular Pathophysiology. *Biomolecules* 2021;11(11). doi:10.3390/biom11111720.

36. **Fan X, Bialecka M, Moustakas I, Lam E, Torrens-Juaneda V, Borggreven NV, Trouw L, Louwe LA, Pilgram GSK, Mei H, van der Westerlaken L, Chuva de Sousa Lopes SM.** Single-cell reconstruction of follicular remodeling in the human adult ovary. *Nat Commun* 2019;10(1):3164.

37. **Liu X, Morency E, Li T, Qin H, Zhang X, Zhang X, Coonrod S.** Role for PADI6 in securing the mRNA-MSY2 complex to the oocyte cytoplasmic lattices. *Cell Cycle* 2017;16(4):360–366.

38. **Auersperg N.** The stem-cell profile of ovarian surface epithelium is reproduced in the oviductal fimbriae, with increased stem-cell marker density in distal parts of the fimbriae. *Int J Gynecol Pathol* 2013;32(5):444–453.

39. **Sweet RA, Nickerson KM, Cullen JL, Wang Y, Shlomchik MJ.** B Cell-Extrinsic Myd88 and Fcer1g Negatively Regulate Autoreactive and Normal B Cell Immune Responses. *J Immunol* 2017;199(3):885–893.

40. **Bebbere D, Masala L, Albertini DF, Ledda S.** The subcortical maternal complex: multiple functions for one biological structure? *J Assist Reprod Genet* 2016;33(11):1431–1438.

41. **Iwata M, Takebayashi T, Ohta H, Alcalde RE, Itano Y, Matsumura T.** Zinc accumulation and metallothionein gene expression in the proliferating epidermis during wound healing in mouse skin. *Histochem Cell Biol* 1999;112(4):283–290.

42. **Gava N, L Clarke C, Bye C, Byth K, deFazio A.** Global gene expression profiles of ovarian surface epithelial cells in vivo. *J Mol Endocrinol* 2008;40(6):281–296.

43. **Paillisson A, Dadé S, Callebaut I, Bontoux M, Dalbiès-Tran R, Vaiman D, Monget P.** Identification, characterization and metagenome analysis of oocyte-specific genes organized in clusters in the mouse genome. *BMC Genomics* 2005;6:76.

44. **Higashi T, Tokuda S, Kitajiri S, Masuda S, Nakamura H, Oda Y, Furuse M.** Analysis of the “angulin” proteins LSR, ILDR1 and ILDR2--tricellulin recruitment, epithelial barrier function and implication in deafness pathogenesis. *J Cell Sci* 2013;126(Pt 4):966–977.

45. **Stables MJ, Shah S, Camon EB, Lovering RC, Newson J, Bystrom J, Farrow S, Gilroy DW.** Transcriptomic analyses of murine resolution-phase macrophages. *Blood* 2011;118(26):e192-208.

46. **Rajkovic A, Lee JH, Yan C, Matzuk MM.** The ret finger protein-like 4 gene, Rfpl4, encodes a putative E3 ubiquitin-protein ligase expressed in adult germ cells. *Mech Dev* 2002;112(1–2):173–177.

47. **Kenngott RA-M, Sauer U, Vermehren M, Sinowatz F.** Expression of Intermediate Filaments and Germ Cell Markers in the Developing Bovine Ovary: An Immunohistochemical and Laser-Assisted Microdissection Study. *Cells Tissues Organs* 2014;200(2):153–170.

48. **Sontheimer RD, Racila E, Racila DM.** C1q: its functions within the innate and adaptive immune responses and its role in lupus autoimmunity. *J Invest Dermatol* 2005;125(1):14–23.

49. **Cai C, Tamai K, Molyneaux K.** KHDC1B is a novel CPEB binding partner specifically expressed in mouse oocytes and early embryos. *Mol Biol Cell* 2010;21(18):3137–3148.

50. **Suto F, Murakami Y, Nakamura F, Goshima Y, Fujisawa H.** Identification and characterization of a novel mouse plexin, plexin-A4. *Mech Dev* 2003;120(3):385–396.

51. **Choi Y, Yuan D, Rajkovic A.** Germ cell-specific transcriptional regulator sohlh2 is essential for early mouse folliculogenesis and oocyte-specific gene expression. *Biol Reprod* 2008;79(6):1176–1182.
