# Supplementary material for: A single-cell atlas of the cycling murine ovary
Source: eLife. 2022 Oct 7;11:e77239. doi: 10.7554/eLife.77239 (PMC9545525; doi:10.7554/eLife.77239)
Supplement: Supplementary file 4. [file elife-77239-supp4.docx]

**Supplementary File 4 - Top 10 markers from each granulosa subclusters**

| **Preantral-Cumulus** | avg_log2FC | **Antral-Mural** | avg_log2FC | **Atretic** | avg_log2FC | **Mitotic** | avg_log2FC |
| --- | --- | --- | --- | --- | --- | --- | --- |
| Gatm (1) | 2.13 | Inhba (2,3) | 2.25 | Ghr | 1.85 | Top2a (4) | 2.30 |
| Kctd14 (5) | 2.05 | Nppc(6–8) | 2.01 | Pik3ip1 (9) | 1.80 | Ube2c (10) | 2.11 |
| Igfbp5 (5) | 1.87 | Mro (11,12) | 2.00 | Cald1 (13) | 1.78 | Racgap1 (14,15) | 1.97 |
| Col18a1 | 1.86 | Nap1l5 (16) | 1.70 | Itih5 | 1.63 | Birc5 | 1.96 |
| Pcsk6 (17) | 1.85 | Hsd17b1 (1) | 1.59 | Cfh | 1.61 | Ccna2 (18,19) | 1.95 |
| Slc18a2 (20) | 1.84 | Slc26a7 (21) | 1.56 | Asb4 | 1.56 | Ccnb2 | 1.86 |
| Fndc5 (22) | 1.79 | Grem1 (3) | 1.54 | Ctgf | 1.51 | Hmgb2 (23) | 1.85 |
| Wt1 (1,24–27) | 1.78 | X1110032F04Rik | 1.53 | Ntn4 (13) | 1.51 | Cdca8 (28) | 1.81 |
| Tmem184a (5) | 1.77 | Cyp19a1 (29) | 1.52 | Cdc42ep3 | 1.44 | Cdk1 (30,31) | 1.80 |
| X1190002N15Rik | 1.74 | Tom1l1 | 1.39 | Fhl1 | 1.40 | Prc1 | 1.75 |

| **Luteinizing mural** | avg_log2FC | **Active CL** | avg_log2FC | **Mitotic-Antral** | avg_log2FC | **Regressing CL** | avg_log2FC |
| --- | --- | --- | --- | --- | --- | --- | --- |
| Tinagl1 (32) | 2.90 | Neat1 (33) | 2.13 | Ccnb2 (34,35) | 1.94 | Ptgfr (13,36) | 2.74 |
| Adamts1 (11,37) | 2.50 | Col3a1 (3) | 2.09 | Ube2c (10) | 1.83 | Efhd1 (38) | 2.50 |
| Mrap (13) | 2.45 | Igfbp7 (39) | 2.03 | Cenpa | 1.75 | S100a6 (40,41) | 2.47 |
| Prss35 (42) | 2.40 | Sfrp4 (43,44) | 1.82 | Top2a (4) | 1.74 | Tnc (11,43) | 2.42 |
| Mt2 (30,45,46) | 2.30 | Cyp11a1 (47) | 1.70 | Racgap1 (14,15) | 1.73 | Lipg (48) | 2.37 |
| Parm1 (49) | 2.26 | Plin4 | 1.71 | Birc5 | 1.72 | Sfrp4 | 2.28 |
| Loxl2 (50) | 2.25 | Onecut2 | 1.71 | Inhbb (1,3) | 1.62 | Lgmn | 2.27 |
| S100a6 (41) | 2.22 | Col1a2 (51) | 1.96 | Ccna2 (18,19) | 1.61 | Star | 2.25 |
| Cemip | 2.20 | Col1a1 (52) | 1.84 | Nap1l5 (16) | 1.60 | Akr1c18 (45,53) | 2.24 |
| Cdkn1a | 2.14 | Gm42669 | 1.75 | Cdca8 (28) | 1.59 | Sgk1 | 2.21 |

Bibliography

1. Fan X, Bialecka M, Moustakas I, Lam E, Torrens-Juaneda V, Borggreven NV, Trouw L, Louwe LA, Pilgram GSK, Mei H, van der Westerlaken L, Chuva de Sousa Lopes SM. Single-cell reconstruction of follicular remodeling in the human adult ovary. *Nat Commun* 2019;10(1):3164.

2. Chen AQ, Wang ZG, Xu ZR, Yu SD, Yang ZG. Analysis of gene expression in granulosa cells of ovine antral growing follicles using suppressive subtractive hybridization. *Anim Reprod Sci* 2009;115(1–4):39–48.

3. Wigglesworth K, Lee K-B, Emori C, Sugiura K, Eppig JJ. Transcriptomic diversification of developing cumulus and mural granulosa cells in mouse ovarian follicles. *Biol Reprod* 2015;92(1):23.

4. Lee JH, Berger JM. Cell Cycle-Dependent Control and Roles of DNA Topoisomerase II. *Genes (Basel)* 2019;10(11). doi:10.3390/genes10110859.

5. Meinsohn M-C, Saatcioglu HD, Wei L, Li Y, Horn H, Chauvin M, Kano M, Nguyen NMP, Nagykery N, Kashiwagi A, Samore WR, Wang D, Oliva E, Gao G, Morris ME, Donahoe PK, Pépin D. Single-cell sequencing reveals suppressive transcriptional programs regulated by MIS/AMH in neonatal ovaries. *Proc Natl Acad Sci U S A* 2021;118(20). doi:10.1073/pnas.2100920118.

6. Zhang M, Su Y-Q, Sugiura K, Xia G, Eppig JJ. Granulosa cell ligand NPPC and its receptor NPR2 maintain meiotic arrest in mouse oocytes. *Science* 2010;330(6002):366–369.

7. Tsuji T, Kiyosu C, Akiyama K, Kunieda T. CNP/NPR2 signaling maintains oocyte meiotic arrest in early antral follicles and is suppressed by EGFR-mediated signaling in preovulatory follicles. *Mol Reprod Dev* 2012;79(11):795–802.

8. Xi G, Wang W, Fazlani SA, Yao F, Yang M, Hao J, An L, Tian J. C-type natriuretic peptide enhances mouse preantral follicle growth. *Reproduction* 2019;157(5):445–455.

9. Piersanti RL, Santos JEP, Sheldon IM, Bromfield JJ. Lipopolysaccharide and tumor necrosis factor-alpha alter gene expression of oocytes and cumulus cells during bovine in vitro maturation. *Mol Reprod Dev* 2019;86(12):1909–1920.

10. Alfieri C, Chang L, Zhang Z, Yang J, Maslen S, Skehel M, Barford D. Molecular basis of APC/C regulation by the spindle assembly checkpoint. *Nature* 2016;536(7617):431–436.

11. Lussier JG, Diouf MN, Lévesque V, Sirois J, Ndiaye K. Gene expression profiling of upregulated mRNAs in granulosa cells of bovine ovulatory follicles following stimulation with hCG. *Reprod Biol Endocrinol* 2017;15(1):88.

12. Rovani MT, Gasperin BG, Ilha GF, Ferreira R, Bohrer RC, Duggavathi R, Bordignon V, Gonçalves PBD. Expression and molecular consequences of inhibition of estrogen receptors in granulosa cells of bovine follicles. *J Ovarian Res* 2014;7:96.

13. Hatzirodos N, Irving-Rodgers HF, Hummitzsch K, Rodgers RJ. Transcriptome profiling of the theca interna from bovine ovarian follicles during atresia. *PLoS One* 2014;9(6):e99706.

14. Wu Y, Lin J, Li X, Han B, Wang L, Liu M, Huang J. Transcriptome profile of one-month-old lambs’ granulosa cells after superstimulation. *Asian-Australas J Anim Sci* 2017;30(1):20–33.

15. Yang X-M, Cao X-Y, He P, Li J, Feng M-X, Zhang Y-L, Zhang X-L, Wang Y-H, Yang Q, Zhu L, Nie H-Z, Jiang S-H, Tian G-A, Zhang X-X, Liu Q, Ji J, Zhu X, Xia Q, Zhang Z-G. Overexpression of Rac GTPase Activating Protein 1 Contributes to Proliferation of Cancer Cells by Reducing Hippo Signaling to Promote Cytokinesis. *Gastroenterology* 2018;155(4):1233-1249.e22.

16. Landry DA, Rossi-Perazza L, Lafontaine S, Sirard M-A. Expression of atresia biomarkers in granulosa cells after ovarian stimulation in heifers. *Reproduction* 2018;156(3):239–248.

17. Diaz FJ, Sugiura K, Eppig JJ. Regulation of Pcsk6 expression during the preantral to antral follicle transition in mice: opposing roles of FSH and oocytes. *Biol Reprod* 2008;78(1):176–183.

18. Blanchard JM. Cyclin A2 transcriptional regulation: modulation of cell cycle control at the G1/S transition by peripheral cues. *Biochem Pharmacol* 2000;60(8):1179–1184.

19. Gong D, Ferrell JEJ. The roles of cyclin A2, B1, and B2 in early and late mitotic events. *Mol Biol Cell* 2010;21(18):3149–3161.

20. Gallardo TD, John GB, Shirley L, Contreras CM, Akbay EA, Haynie JM, Ward SE, Shidler MJ, Castrillon DH. Genomewide discovery and classification of candidate ovarian fertility genes in the mouse. *Genetics* 2007;177(1):179–194.

21. Richards JS, Fan H-Y, Liu Z, Tsoi M, Laguë M-N, Boyer A, Boerboom D. Either Kras activation or Pten loss similarly enhance the dominant-stable CTNNB1-induced genetic program to promote granulosa cell tumor development in the ovary and testis. *Oncogene* 2012;31(12):1504–1520.

22. Luo Y, Qiao X, Ma Y, Deng H, Xu CC, Xu L. Irisin deletion induces a decrease in growth and fertility in mice. *Reprod Biol Endocrinol* 2021;19(1):22.

23. Pallier C, Scaffidi P, Chopineau-Proust S, Agresti A, Nordmann P, Bianchi ME, Marechal V. Association of chromatin proteins high mobility group box (HMGB) 1 and HMGB2 with mitotic chromosomes. *Mol Biol Cell* 2003;14(8):3414–3426.

24. Zhao Z-H, Ma J-Y, Meng T-G, Wang Z-B, Yue W, Zhou Q, Li S, Feng X, Hou Y, Schatten H, Ou X-H, Sun Q-Y. Single-cell RNA sequencing reveals the landscape of early female germ cell development. *FASEB J* 2020;34(9):12634–12645.

25. Chun SY, McGee EA, Hsu SY, Minami S, LaPolt PS, Yao HH, Bahr JM, Gougeon A, Schomberg DW, Hsueh AJ. Restricted expression of WT1 messenger ribonucleic acid in immature ovarian follicles: uniformity in mammalian and avian species and maintenance during reproductive senescence. *Biol Reprod* 1999;60(2):365–373.

26. Gao F, Zhang J, Wang X, Yang J, Chen D, Huff V, Liu Y-X. Wt1 functions in ovarian follicle development by regulating granulosa cell differentiation. *Hum Mol Genet* 2014;23(2):333–341.

27. Park M, Choi Y, Choi H, Roh J. Wilms’ tumor suppressor gene (WT1) suppresses apoptosis by transcriptionally downregulating BAX expression in immature rat granulosa cells. *J Ovarian Res* 2014;7:118.

28. Gassmann R, Carvalho A, Henzing AJ, Ruchaud S, Hudson DF, Honda R, Nigg EA, Gerloff DL, Earnshaw WC. Borealin: a novel chromosomal passenger required for stability of the bipolar mitotic spindle. *J Cell Biol* 2004;166(2):179–191.

29. Bertolin K, Meinsohn M-C, Suzuki J, Gossen J, Schoonjans K, Duggavathi R, Murphy BD. Ovary-specific depletion of the nuclear receptor Nr5a2 compromises expansion of the cumulus oophorus but not fertilization by intracytoplasmic sperm injection. *Biol Reprod* 2017;96(6):1231–1243.

30. Wang S, Liu W, Pang X, Dai S, Liu G. The Mechanism of Melatonin and Its Receptor MT2 Involved in the Development of Bovine Granulosa Cells. *Int J Mol Sci* 2018;19(7). doi:10.3390/ijms19072028.

31. Jones MC, Zha J, Humphries MJ. Connections between the cell cycle, cell adhesion and the cytoskeleton. *Philos Trans R Soc Lond B Biol Sci* 2019;374(1779):20180227.

32. Akaiwa M, Fukui E, Matsumoto H. Tubulointerstitial nephritis antigen-like 1 deficiency alleviates age-dependent depressed ovulation associated with ovarian collagen deposition in mice. *Reprod Med Biol* 2020;19(1):50–57.

33. Nakagawa S, Shimada M, Yanaka K, Mito M, Arai T, Takahashi E, Fujita Y, Fujimori T, Standaert L, Marine J-C, Hirose T. The lncRNA Neat1 is required for corpus luteum formation and the establishment of pregnancy in a subpopulation of mice. *Development* 2014;141(23):4618–4627.

34. Bellanger S, de Gramont A, Sobczak-Thépot J. Cyclin B2 suppresses mitotic failure and DNA re-replication in human somatic cells knocked down for both cyclins B1 and B2. *Oncogene* 2007;26(51):7175–7184.

35. Huang Y, Sramkoski RM, Jacobberger JW. The kinetics of G2 and M transitions regulated by B cyclins. *PLoS One* 2013;8(12):e80861.

36. Berisha B, Rodler D, Schams D, Sinowatz F, Pfaffl MW. Prostaglandins in Superovulation Induced Bovine Follicles During the Preovulatory Period and Early Corpus Luteum. *Front Endocrinol (Lausanne)* 2019;10:467.

37. Sayasith K, Lussier J, Sirois J. Molecular characterization and transcriptional regulation of a disintegrin and metalloproteinase with thrombospondin motif 1 (ADAMTS1) in bovine preovulatory follicles. *Endocrinology* 2013;154(8):2857–2869.

38. Kfir S, Basavaraja R, Wigoda N, Ben-Dor S, Orr I, Meidan R. Genomic profiling of bovine corpus luteum maturation. *PLoS One* 2018;13(3):e0194456.

39. Tamura K, Matsushita M, Endo A, Kutsukake M, Kogo H. Effect of insulin-like growth factor-binding protein 7 on steroidogenesis in granulosa cells derived from equine chorionic gonadotropin-primed immature rat ovaries. *Biol Reprod* 2007;77(3):485–491.

40. Duan WR, Parmer TG, Albarracin CT, Zhong L, Gibori G. PRAP, a prolactin receptor associated protein: its gene expression and regulation in the corpus luteum. *Endocrinology* 1997;138(8):3216–3221.

41. Hanaue M, Miwa N, Takamatsu K. Immunohistochemical Characterization of S100A6 in the Murine Ovary. *Acta Histochem Cytochem* 2012;45(1):9–14.

42. Wahlberg P, Nylander A, Ahlskog N, Liu K, Ny T. Expression and localization of the serine proteases high-temperature requirement factor A1, serine protease 23, and serine protease 35 in the mouse ovary. *Endocrinology* 2008;149(10):5070–5077.

43. Hernandez-Gonzalez I, Gonzalez-Robayna I, Shimada M, Wayne CM, Ochsner SA, White L, Richards JS. Gene expression profiles of cumulus cell oocyte complexes during ovulation reveal cumulus cells express neuronal and immune-related genes: does this expand their role in the ovulation process? *Mol Endocrinol* 2006;20(6):1300–1321.

44. Zamberlam G, Lapointe E, Abedini A, Rico C, Godin P, Paquet M, DeMayo FJ, Boerboom D. SFRP4 Is a Negative Regulator of Ovarian Follicle Development and Female Fertility. *Endocrinology* 2019;160(7):1561–1572.

45. He W, Gauri M, Li T, Wang R, Lin S-X. Current knowledge of the multifunctional 17β-hydroxysteroid dehydrogenase type 1 (HSD17B1). *Gene* 2016;588(1):54–61.

46. Wang SJ, Liu WJ, Wu CJ, Ma FH, Ahmad S, Liu BR, Han L, Jiang XP, Zhang SJ, Yang LG. Melatonin suppresses apoptosis and stimulates progesterone production by bovine granulosa cells via its receptors (MT1 and MT2). *Theriogenology* 2012;78(7):1517–1526.

47. Irving-Rodgers HF, Harland ML, Sullivan TR, Rodgers RJ. Studies of granulosa cell maturation in dominant and subordinate bovine follicles: novel extracellular matrix focimatrix is co-ordinately regulated with cholesterol side-chain cleavage CYP11A1. *Reproduction* 2009;137(5):825–834.

48. Lee-Thacker S, Choi Y, Taniuchi I, Takarada T, Yoneda Y, Ko C, Jo M. Core Binding Factor β Expression in Ovarian Granulosa Cells Is Essential for Female Fertility. *Endocrinology* 2018;159(5):2094–2109.

49. Park JY, Jang H, Curry TE, Sakamoto A, Jo M. Prostate androgen-regulated mucin-like protein 1: a novel regulator of progesterone metabolism. *Mol Endocrinol* 2013;27(11):1871–1886.

50. Baufeld A, Koczan D, Vanselow J. Induction of altered gene expression profiles in cultured bovine granulosa cells at high cell density. *Reprod Biol Endocrinol* 2017;15(1):3.

51. Hodgkinson K, Forrest LA, Vuong N, Garson K, Djordjevic B, Vanderhyden BC. GREB1 is an estrogen receptor-regulated tumour promoter that is frequently expressed in ovarian cancer. *Oncogene* 2018;37(44):5873–5886.

52. Wagner M, Yoshihara M, Douagi I, Damdimopoulos A, Panula S, Petropoulos S, Lu H, Pettersson K, Palm K, Katayama S, Hovatta O, Kere J, Lanner F, Damdimopoulou P. Single-cell analysis of human ovarian cortex identifies distinct cell populations but no oogonial stem cells. *Nat Commun* 2020;11(1):1147.

53. Kapfhamer J, Waite C, Ascoli M. The Gα(q/11)-provoked induction of Akr1c18 in murine luteal cells is mediated by phospholipase C. *Mol Cell Endocrinol* 2018;470:179–187.
